# Can Artificial Intelligence Transform Early Warning for Antimicrobial-Resistant Outbreak Clones? Approaches, Gaps, and Opportunities: A Scoping Review

**DOI:** 10.3390/antibiotics15060599

**Published:** 2026-06-12

**Authors:** Adriana Antonina Tempesta, Eleonora Chines, Ludovica Boscarelli, Matteo Francesco Parisi, Lorenzo Marcoccia, Antonino Capillo, Maria Lina Mezzatesta, Caterina Ledda, Marco Chessari, Viviana Cafiso

**Affiliations:** 1 Department of Biomedical and Biotechnological Sciences, University of Catania, 95123 Catania, Italy; adriana.tempesta@phd.unict.it (A.A.T.); eleonora.chines01@universitadipavia.it (E.C.); ludovica.boscarelli@studium.unict.it (L.B.); m.parisi@studium.unict.it (M.F.P.); mezzate@unict.it (M.L.M.); 2 PhD National Program in One Health Approaches to Infectious Diseases and Life Science Research, Department of Public Health, Experimental, and Forensic Medicine, University of Pavia, 27100 Pavia, Italy; 3 Unit of AI and Computer Systems, Department of Engineering, Università Campus Bio-Medico di Roma, 00128 Rome, Italy; l.marcoccia@teleconsys.it; 4 HorAlzon Lab, Teleconsys SpA, 00144 Rome, Italy; a.capillo@teleconsys.it (A.C.); m.chessari@teleconsys.it (M.C.); 5 Occupational Medicine, Department of Clinical and Experimental Medicine, University of Catania, 95123 Catania, Italy; caterina.ledda@unict.it

**Keywords:** antimicrobial resistance, artificial intelligence, genomic surveillance, whole-genome sequencing, outbreak detection, infection prevention and control

## Abstract

**Background/Objectives:** Antimicrobial resistance (AMR), driven by high-risk bacterial pathogens, is a major healthcare threat. This scoping review mapped artificial intelligence/machine learning (AI/ML) and computational approaches integrated with whole-genome sequencing (WGS), genomic surveillance, rapid typing, epidemiological data, or clinical metadata for early warning of AMR outbreak clones. **Methods:** Following PRISMA-ScR guidance and the Population–Concept–Context (PCC) framework, PubMed/MEDLINE, Scopus, and Web of Science were searched for English-language studies published between 2010 and 2026. Eligible studies addressed AI/ML or computational approaches for AMR outbreak detection, clone surveillance, transmission analysis, or infection prevention and control (IPC). **Results:** Thirty-eight studies were grouped into five domains: genomic surveillance; rapid typing; resistance, risk-factor, and lineage prediction; transmission reconstruction; and IPC-oriented genomic epidemiology. AI/ML supported automation, isolate prioritization, typing triage, prediction, transmission modelling, and electronic health record (EHR)-linked route identification. **Conclusions:** AI/ML may enhance WGS-based AMR surveillance, but validation, dataset dependence, heterogeneity, and limited IPC outcome reporting remain key gaps.

## 1. Introduction

Antimicrobial resistance (AMR) remains a major global health threat, with recent estimates indicating that bacterial AMR was associated with 4.71 million deaths in 2021, including 1.14 million deaths directly attributable to resistant infections [1]. The public health relevance of this threat is reflected in the WHO 2024 Bacterial Priority Pathogens List, which prioritizes several multidrug-resistant organisms frequently involved in healthcare-associated infections, including carbapenem-resistant *Klebsiella pneumoniae* (CRKP), carbapenem-resistant *Acinetobacter baumannii* (CRAB), carbapenem-resistant *Escherichia coli* (CREC), methicillin-resistant *Staphylococcus aureus* (MRSA), and vancomycin-resistant *Enterococcus faecium* (VRE) [2,3]. In healthcare settings, these pathogens are of particular concern because they can cause severe infections, spread across wards or institutions, and limit available therapeutic options.

The dissemination of AMR in healthcare environments is often driven by international high-risk clones and mobile genetic elements. High-risk lineages provide successful genomic backgrounds for the acquisition, maintenance, and spread of resistance determinants [4]. Examples include *E. coli* ST131, frequently associated with extended-spectrum β-lactamases, and *K. pneumoniae* ST258, which has been a major driver of the global dissemination of KPC carbapenemases (KPC)-producing strains [5,6,7]. In addition to clonal expansion, plasmid-mediated dissemination contributes to the spread of carbapenemase genes and other resistance determinants across bacterial species and healthcare networks [5]. These features make AMR outbreak clones difficult to detect, contain, and monitor using conventional microbiological and epidemiological approaches alone.

Whole-genome sequencing (WGS) and genomic surveillance have transformed the investigation of healthcare-associated outbreaks by enabling high-resolution assessment of genetic relatedness, cluster confirmation or refutation, and distinction between local transmission and multiple introductions [8,9]. When integrated with epidemiological information, patient movement data, environmental sampling, or infection prevention and control (IPC) workflows, WGS can uncover occult transmission routes and support targeted interventions [10,11,12]. However, routine implementation remains challenged by sequencing capacity, turnaround time, data interpretation, costs, and the need to translate genomic findings into actionable IPC decisions.

Artificial intelligence (AI), machine learning (ML), and advanced computational approaches may help address some of these implementation challenges by supporting automation, prioritization, prediction, transmission modelling, and integration of heterogeneous surveillance data [13,14,15]. Although AI-focused AMR literature has often emphasized phenotype prediction, resistance and virulence determinant prioritization, omics-based target discovery, and antimicrobial development [13,14,15], this review focuses on their use as operational layers within outbreak surveillance. In this context, AI/ML and computational approaches may complement WGS by supporting earlier recognition, isolate triage, automated genomic interpretation, transmission reconstruction, plasmid-aware genomic epidemiology, and rapid typing-based prioritization for genomic confirmation.

Given this methodological heterogeneity, a scoping review approach was appropriate to summarize how AI/ML and advanced computational approaches are being used to strengthen AMR outbreak surveillance in healthcare settings. This review aimed to identify evidence gaps and highlight implementation opportunities for translating genomic and computational outputs into actionable IPC intelligence.

## 2. Materials and Methods

### 2.1. Study Design

This scoping review was conducted in accordance with the Preferred Reporting Items for Systematic Reviews and Meta-Analyses [16] extension for Scoping Reviews (PRISMA-ScR) (Supplementary File S1). This approach was considered appropriate for mapping an emerging field that combines artificial intelligence, machine learning, advanced computational approaches, genomic surveillance, antimicrobial resistance, and healthcare outbreak investigation.

The review question was structured according to the Population–Concept–Context (PCC) framework as follows: “How are artificial intelligence, machine learning, and advanced computational approaches integrated with whole-genome sequencing and genomic surveillance to support the early detection, characterization, and investigation of antimicrobial-resistant outbreak clones in healthcare settings?”

The PCC framework used to define the scope of the review and guide the eligibility criteria is reported in Table 1. The protocol was retrospectively registered in the Open Science Framework on 4 June 2026 (https://doi.org/10.17605/OSF.IO/56XBK). The review question, eligibility criteria, and data-charting form had been defined before the final synthesis of results.

### 2.2. Search Strategy

The literature search was conducted in PubMed/MEDLINE, Scopus, and Web of Science for studies published between 2010 and 2026. Searches were limited to English-language articles, with no geographical restrictions. Search strategies were developed from the review question and PCC framework using Boolean combinations of terms related to artificial intelligence and machine learning, whole-genome sequencing and genomic surveillance, antimicrobial resistance, outbreak investigation, transmission, clones, and healthcare-associated surveillance.

In PubMed/MEDLINE, three complementary search strings were used to increase sensitivity, and records retrieved from these searches were deduplicated before inclusion in the final PubMed/MEDLINE count. Scopus and Web of Science were searched using database-specific adaptations of the main strategy. An additional Web of Science sensitivity search was performed, yielding one further eligible record. The full database-specific search strategies, applied limits, record counts, and deduplication steps are reported in Supplementary Table S1.

### 2.3. Inclusion and Exclusion Criteria

Inclusion and exclusion criteria were defined a priori according to the review question and PCC framework. Studies were included if they addressed bacterial antimicrobial-resistant pathogens relevant to healthcare-associated transmission and investigated AI/ML or advanced computational, statistical, or integrative approaches. Eligible full-text original studies, including short communications, had to combine these approaches with WGS/genomic surveillance, rapid typing, or epidemiological and/or clinical data, and had to support the detection, characterization, transmission reconstruction, or investigation of AMR outbreak clones. Studies were excluded if they were reviews, editorials, commentaries, conference abstracts with insufficient methodological detail, non-bacterial studies, non-English articles, or not available in full text. Studies focused only on antimicrobial discovery, therapeutic target identification, antimicrobial peptide design, AMR phenotype/MIC prediction unrelated to genomic surveillance, or exclusively non-healthcare environmental, food, veterinary, or One Health contexts were also excluded.

### 2.4. Study Selection and Data Extraction

All retrieved records were imported into reference management software (EndNote version 2025.3), and duplicates were removed. Two independent reviewers screened titles and abstracts according to the predefined inclusion and exclusion criteria. Records considered potentially eligible were assessed through full-text review. Disagreements were resolved by discussion, and a third reviewer was consulted when consensus could not be reached. Data were extracted using a standardized data-charting form developed for this review. The extracted information included author, year of publication, country, study design, healthcare or surveillance setting, bacterial pathogen, antimicrobial resistance profile, genomic method, AI/ML or computational approach, data sources, main outcome, relevance to outbreak detection or transmission investigation, and evidence domain. The data-charting form was refined iteratively during the extraction process to ensure consistency with the objectives of the review.

### 2.5. Synthesis of Results

The included studies were synthesized using a structured evidence-mapping approach. Extracted data were compared across pathogens, healthcare settings, genomic or typing methods, AI/ML or computational approaches, data sources, and reported surveillance or outbreak-investigation outcomes. Studies were grouped into evidence domains according to their primary contribution to the review question. When studies addressed more than one area, they were assigned to the domain that best reflected their main objective or most relevant contribution.

In addition to descriptive mapping, a domain-level interpretive synthesis was performed to identify recurrent methodological constraints, implementation issues, evidence gaps, and opportunities for IPC translation. This synthesis was not intended as a formal risk-of-bias assessment or quantitative quality appraisal, but as a structured critical mapping of evidence maturity within each domain.

## 3. Results

### 3.1. Study Selection

**Figure 1:**
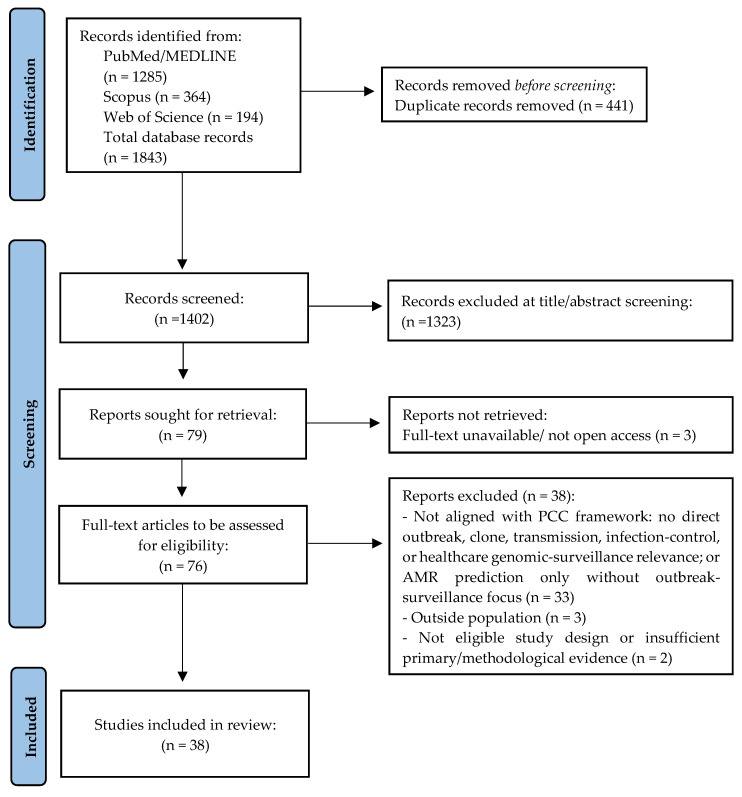
PRISMA-ScR flow diagram of the study selection process.

The search strategy identified 1843 records from PubMed/MEDLINE (*n* = 1285), Scopus (*n* = 364), and Web of Science (*n* = 194). The Web of Science count included 193 records from the main search and one additional eligible record identified through a sensitivity search. After 441 duplicates were removed, 1402 records were screened by title and abstract, of which 1323 were excluded. Seventy-nine reports were sought for full-text retrieval. Three reports could not be retrieved, and 76 full-text reports were assessed for eligibility. Of these, 38 were excluded, and 38 studies were included in the final scoping review (Figure 1).

### 3.2. General Characteristics of Included Studies

The 38 included studies addressed AI/ML and advanced computational approaches applied to genomic surveillance, outbreak investigation, rapid typing, and transmission analysis of antimicrobial-resistant bacterial pathogens in healthcare settings [17,18,19,20,21,22,23,24,25,26,27,28,29,30,31,32,33,34,35,36,37,38,39,40,41,42,43,44,45,46,47,48,49,50,51,52,53,54]. The most frequently investigated pathogens were *K. pneumoniae*, *S. aureus*/MRSA, *A. baumannii*, *Pseudomonas aeruginosa*, *E. faecium*/VRE, *E. coli*, *Enterobacter cloacae* complex, and other *Enterobacterales.* The studies covered neonatal and pediatric units, intensive care units, tertiary hospitals, endoscopy-associated outbreaks, regional or national surveillance programmes, and public health outbreak investigations. Genomic and typing approaches included WGS, cgMLST, SNP-based analysis, long-read sequencing, plasmid-level analysis, FTIR/ATR-FTIR, MALDI-TOF MS, SERS, and IR-Biotyper workflows. These were combined with ML classification, predictive modelling, automated genomic interpretation, EHR-based data integration, Bayesian and phylodynamic approaches, and other computational methods. The main characteristics of the included studies are summarized in Table 2.

### 3.3. Evidence Domains

**Figure 2:**
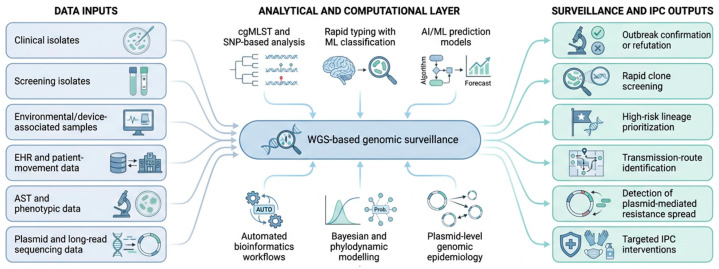
Conceptual workflow of AI-assisted antimicrobial resistance outbreak surveillance. Clinical, microbiological, genomic, epidemiological, environmental, and electronic health record-derived data feed into whole-genome sequencing-based surveillance supported by artificial intelligence, machine learning, and computational methods. These approaches can generate actionable outputs, including outbreak confirmation, rapid clone screening, transmission-route identification, detection of plasmid-mediated resistance spread, and targeted infection prevention and control interventions.

Five evidence domains were identified according to the main operational contribution of each study: genomic surveillance for outbreak detection and investigation; rapid typing and clone screening; prediction of resistance, risk factors, and high-risk lineages; transmission reconstruction and outbreak dynamics; and IPC-oriented integrated genomic epidemiology. These domains reflect a common operational pathway in which multisource data are progressively translated into surveillance and infection prevention outputs. This pathway is summarized in Figure 2. The following sections present these domains, with studies cited according to their primary contribution.

#### 3.3.1. Genomic Surveillance for Outbreak Detection and Investigation

Eight studies focused on genomic surveillance for outbreak detection and investigation [17,18,19,20,21,22,23,24]. Four studies by Sundermann et al. evaluated the Enhanced Detection System for Healthcare-Associated Transmission (EDS-HAT) and related computational workflows across retrospective outbreak detection, hospital-wide surveillance, AI-assisted route identification, and real-time implementation [17,18,19,20]. In the initial retrospective analysis, WGS detected an unrecognized *P. aeruginosa* gastroscope-associated outbreak, while ML analysis of electronic health record (EHR) data supported gastroscopy as the likely transmission route [17]. In hospital-wide surveillance, EDS-HAT identified genetically related clusters and transmission routes that had not been recognized by conventional IPC practice [18]. A subsequent study explicitly evaluated AI-assisted analysis of EHR-derived exposures to identify transmission routes missed by manual review [19]. Real-time implementation of EDS-HAT supported IPC interventions and assessment of surveillance impact [20]. A complementary automation-focused study by Raven et al. evaluated a cloud-based platform for MRSA genomic outbreak analysis [21]. The platform showed concordance with manual analysis for key genomic tasks, including species identification, *mec* detection, sequence type assignment, and cluster detection, supporting automated confirmation or refutation of suspected MRSA outbreaks [21]. Two studies extended genomic surveillance to neonatal screening settings [22,23]. Price et al. used WGS of Gram-negative isolates from routine neonatal screening to identify putative transmission clusters and possible AMR gene movement not detected by routine screening alone [23]. Böhne et al. integrated WGS, epidemiological analysis, and exploratory ML models in a *K. pneumoniae* NICU surveillance programme to identify high-risk patient groups and support risk-adapted IPC [22]. Ross et al. addressed surveillance scalability from a different angle, using routine AST data and ML models to predict *E. coli* ST131 clade C membership when WGS was unavailable for all isolates [24].

The evidence base is mainly informed by four studies from a single research group (Sundermann et al.) at one institution, which may limit generalizability given the reliance of EDS-HAT on local EHR infrastructure and pathogen epidemiology [17,18,19,20]. ML-derived transmission routes should be interpreted as associative and require further validation, and benchmarking against manual review or manual analysis—as in Raven et al.—may be limited in the absence of an independent epidemiological reference standard [17,18,19,20,21]. The neonatal studies involve small single-unit cohorts with limited statistical power, and Böhne et al.’s explicitly exploratory ML models require further validation before clinical implementation [22,23]. Across all eight studies, limited patient-level outcome data make it difficult to assess the clinical effectiveness of these systems beyond cluster detection metrics [17,18,19,20,21,22,23,24].

Despite these limitations, these studies collectively establish that WGS integrated with ML-driven EHR analysis can systematically uncover transmission events and high-risk clusters invisible to conventional IPC surveillance. The demonstrated progression of EDS-HAT from retrospective detection to real-time operational deployment, alongside scalable alternatives such as automated genomic platforms and phenotypic surrogates for WGS-unavailable settings, provides a credible and increasingly actionable framework for next-generation healthcare-associated infection surveillance.

#### 3.3.2. Rapid Typing and Clone Screening

Nine studies evaluated rapid typing and clone screening as alternatives or complements to WGS-based outbreak investigation [25,26,27,28,29,30,31,32,33]. Five studies assessed FTIR, ATR-FTIR, or IR-Biotyper workflows for rapid bacterial typing [25,26,27,32,33]. These studies focused mainly on *K. pneumoniae*, *E. cloacae* complex, and other Gram-negative bacteria in clinical or surveillance settings. Supervised ML models, spectral clustering, and species-specific thresholds were used to improve classification, support rapid clone screening, and prioritize isolates for genomic confirmation. In these studies, WGS or cgMLST remained the reference approach for definitive outbreak confirmation [25,26,27,32,33]. Three studies used MALDI-TOF MS-based approaches for rapid clone or resistance-mechanism screening [28,29,31]. Together, they applied spectral classification to CRKP, *E. coli* ST131, and MDR *P. aeruginosa* ST175 outbreak isolates, using ML models to screen high-risk lineages or resistance-associated groups. Reported performance varied across lineages and settings [28,29,31]. A further proof-of-concept study explored SERS combined with ML for *K. pneumoniae* typing, using supervised classification to assess ST-level discrimination in a small strain collection [30].

The nine studies are methodologically heterogeneous, limiting aggregate evaluation, with variation in spectral platforms, preprocessing pipelines, ML algorithms, and similarity thresholds, rendering performance metrics non-comparable across studies. The predominant focus on *K. pneumoniae* and *E. cloacae* complex limits generalizability to other clinically relevant pathogens. A key methodological consideration is that these rapid typing approaches were often evaluated against WGS or cgMLST reference standards; future studies should also assess their operational value against clinical or epidemiological outcomes, rather than genomic concordance alone. Prospective validation of fixed models in independent cohorts was limited, and implementation-critical data, such as turnaround time, cost, and false-positive consequences for cohorting decisions, were rarely reported. The SERS proof-of-concept study should be interpreted as preliminary and distinct from more developed rapid-typing workflows.

Despite this heterogeneity, these studies collectively support the concept of a tiered surveillance model in which spectral platforms serve as intelligent, high-throughput triage tools to prioritise isolates for selective WGS confirmation. The growing adoption of supervised learning frameworks and species-specific thresholds signals methodological maturation, and the demonstrated discriminatory capacity of FTIR and MALDI-TOF MS for high-risk lineages offers a pragmatic, laboratory-embedded solution for settings where universal WGS access remains limited.

#### 3.3.3. Prediction of Resistance, Risk Factors, and High-Risk Lineages

Seven studies examined the prediction of resistance, risk factors, and high-risk lineages in healthcare-associated AMR surveillance [34,35,36,37,38,39,40]. Two studies combined WGS and ML to assess dissemination risk or misleading surveillance signals in neonatal settings [34,35]. Liu et al. integrated WGS, selected long-read sequencing, clinical metadata, and random forest models to identify factors associated with CRKP persistence and outbreak-associated genotypes, including clonal spread, healthcare-group-associated transmission, and carbapenemase-carrying plasmids [34]. Sawhney et al. used WGS and a random forest classifier during a suspected MRSA pseudo-outbreak, distinguishing BORSA from MSSA and refuting clonal MRSA expansion in a NICU [35].

Beyond neonatal outbreak or pseudo-outbreak settings, four studies extended this domain to *S. aureus*/MRSA genomic surveillance [36,37,38,39]. They applied WGS-derived genomic features and supervised ML to predict antimicrobial resistance, susceptibility phenotypes, or resistance-associated lineage structure, using k-mers, cgMLST allelic profiles, AMR gene patterns, MIC data, random forest, SVM, XGBoost, Elastic Net, and PLS. These studies linked resistance phenotypes with lineage structure, dominant MRSA backgrounds, and large-scale β-lactam/*mecA* resistance dynamics, with variable performance for complex β-lactam phenotypes [36,37,38,39]. In the same MRSA surveillance area, Mauffrey et al. used WGS, pan-genome analysis, GWAS, time-scaled haplotypic density, and random forest models to study ST228 SCCmec-I MRSA after a major nosocomial outbreak [40]. The analysis identified genomic features associated with declining epidemicity and monitored the trajectory of this epidemic clone [40].

This group of seven studies was methodologically diverse, encompassing neonatal pseudo-outbreak investigation, large-scale resistance phenotype prediction, and post-outbreak clone trajectory analysis, which limits direct comparison across studies.

The two neonatal studies address distinct clinical questions—one clonal persistence, one pseudo-outbreak refutation—and neither is directly generalizable beyond its specific institutional context. The four MRSA/*S. aureus* genomic prediction studies employ a wide array of feature types and algorithms (k-mers, cgMLST profiles, AMR gene patterns, MIC data, random forest, SVM, XGBoost, Elastic Net, PLS) without apparent methodological consensus, making cross-study synthesis challenging. The acknowledged variable performance for complex β-lactam phenotypes is a clinically significant limitation that requires further investigation. Mauffrey et al.’s sophisticated genomic epidemiology study of ST228 SCCmec-I MRSA, while analytically rich, is inherently retrospective and single-outbreak in scope, limiting its broader applicability [40].

Nevertheless, the studies collectively illustrate the expanding analytical repertoire available for AMR surveillance, from pseudo-outbreak discrimination and resistance phenotype prediction to post-epidemic clone monitoring. The successful application of ML to refute clonal expansion, identify plasmid-mediated transmission drivers, and track epidemic trajectory demonstrates that genomic-ML integration can address clinically actionable questions across the full outbreak lifecycle, from early detection to resolution.

#### 3.3.4. Transmission Reconstruction and Outbreak Dynamics

Four studies investigated transmission reconstruction and outbreak dynamics using WGS combined with Bayesian, stochastic, or phylodynamic approaches [41,42,43,44]. Three studies applied Bayesian or stochastic modelling to hospital outbreak investigations [41,42,43]. Fujikura et al. used WGS and Bayesian modelling to reconstruct transmission during a vancomycin-resistant *Enterococcus* (VRE) outbreak in a Japanese tertiary hospital; by integrating genomic variants, sampling time, and patient exposure data, the model inferred transmission direction, identified spreaders, and suggested environmental transmission [41]. Shimizu et al. investigated a suspected outbreak of carbapenem-resistant *P. aeruginosa* (CRPA) in a Japanese tertiary children’s hospital; hybrid WGS showed that isolates belonged to multiple sequence types rather than a single monoclonal outbreak, and Bayesian inference applied to a subset of related isolates did not establish a definitive transmission pathway [42]. Silvotti et al. used WGS and Bayesian transmission-network reconstruction to investigate KPC-producing *K. pneumoniae* in an Italian neurorehabilitation unit, identifying multiple independent introductions, secondary transmission chains, and weaknesses in admission screening, surveillance, and staff training [43]. A fourth study focused on nanopore-based phylodynamic outbreak reconstruction [44]. Steinig et al. combined low-coverage nanopore sequencing, random forest-based refinement of SNP calls, and Bayesian birth–death skyline modelling to assess MRSA ST93-MRSA-IV outbreak reconstruction in remote northern Australia and Papua New Guinea [44].

The four studies are methodologically coherent in their shared reliance on WGS-informed probabilistic modelling but collectively represent a small and geographically dispersed evidence base spanning distinct pathogens, healthcare settings, and epidemiological contexts that limits synthesis. The Bayesian and stochastic approaches, while analytically rigorous, are computationally demanding and require dense epidemiological metadata—patient movement, exposure timing, environmental sampling—that may not be routinely available in most healthcare settings, which may limit real-world transferability. Shimizu et al. did not establish a definitive transmission pathway despite sophisticated modelling, highlighting an important limitation: genomic resolution alone may not resolve transmission ambiguity when multiple sequence types co-circulate, or sampling density is insufficient.

Nonetheless, these studies demonstrate that probabilistic transmission reconstruction can generate actionable IPC insights beyond what conventional epidemiology detects, including identification of spreaders, independent introduction events, and screening gaps. The inclusion of a low-coverage nanopore-based workflow is particularly noteworthy, as it suggests that phylodynamic outbreak reconstruction may be achievable in resource-limited or remote settings where short-read WGS infrastructure is unavailable, broadening the potential reach of genomic-informed outbreak response.

#### 3.3.5. IPC-Oriented Integrated Genomic Epidemiology

Ten studies focused on IPC-oriented integrated genomic epidemiology, using WGS or long-read sequencing to support outbreak investigation, ward-based surveillance, environmental or device-associated transmission assessment, plasmid tracking, and infection-control decision-making [45,46,47,48,49,50,51,52,53,54]. Several studies used WGS-based genomic epidemiology to define the clonal structure and dissemination of high-risk AMR pathogens within hospitals or across regional healthcare networks [45,46,48,53]. These studies investigated MDR *K. pneumoniae*, XDR carbapenem-resistant *A. baumannii* (CRAB), vancomycin-resistant *E. faecium*, and 3GC- or carbapenem-resistant *K. pneumoniae.* Across these settings, WGS, core-genome phylogeny, SNP analysis, comparative genomics, and regional phylogenetic contextualization distinguished multiple outbreak lineages, defined high-risk clonal groups, and mapped intra- or inter-hospital transmission patterns [45,46,48,53]. Other studies extended IPC-oriented genomic epidemiology to environmental and device-associated transmission [47,49,50]. Two CRAB studies in Chinese ICUs combined clinical and environmental sampling to assess patient–environment relatedness, environmental contamination, medical-equipment involvement, and possible inter-ICU transmission [47,49]. Cissé et al. combined epidemiological reconstruction, FTIR, and WGS to investigate *P. aeruginosa* ST1320 transmission associated with contaminated duodenoscopes [50]. Plasmid-level and long-read sequencing approaches further expanded the scope of IPC-oriented surveillance beyond strain relatedness [51,52,54]. Jalal et al. and Leder et al. combined WGS, plasmid reconstruction, and patient-movement data to distinguish clonal spread from plasmid-mediated carbapenemase dissemination across unrelated lineages or species [51,52]. Landman et al. evaluated Nanopore long-read sequencing for MDRO genomic surveillance, molecular typing, AMR gene detection, plasmid-replicon detection, and outbreak analysis [54].

The ten studies are clinically diverse but methodologically uneven, spanning single-ward outbreak investigations, regional network analyses, environmental sampling, and plasmid-level surveillance across distinct pathogens and healthcare systems, which limits coherent synthesis. Studies conducted in Chinese ICUs and other single-institution settings are subject to local epidemiological confounding, and the absence of standardized epidemiological outcome measures across studies makes it difficult to assess whether genomic findings translated into effective IPC interventions. The two CRAB environmental studies rely on opportunistic sampling frameworks, which may not fully represent the underlying contamination burden. Plasmid reconstruction approaches, while analytically valuable, remain technically demanding and are not yet standardized across platforms. Landman et al.’s Nanopore-based evaluation, though promising, represents a single-centre feasibility assessment rather than a validated clinical deployment.

Collectively, these studies demonstrate the expanding operational scope of WGS-based genomic epidemiology in IPC, moving beyond strain-level cluster detection to encompass environmental reservoirs, device-associated transmission, inter-hospital spread, and horizontal gene transfer via plasmid tracking. The integration of long-read sequencing and plasmid reconstruction into routine surveillance workflows represents a particularly significant advance, enabling discrimination between clonal expansion and plasmid-mediated AMR dissemination—a distinction with direct consequences for targeted IPC intervention and regional containment strategies.

## 4. Discussion

This scoping review mapped 38 studies evaluating AI/ML and advanced computational approaches integrated with genomic surveillance, WGS, rapid typing, epidemiological data, or clinical metadata for the detection, characterization, and investigation of antimicrobial-resistant outbreak clones in healthcare settings [17,18,19,20,21,22,23,24,25,26,27,28,29,30,31,32,33,34,35,36,37,38,39,40,41,42,43,44,45,46,47,48,49,50,51,52,53,54]. Overall, the evidence indicates that these approaches are not replacing WGS-based surveillance, but are expanding its operational value by improving triage, scalability, automation, transmission interpretation, and integration with IPC-relevant data. Across the included studies, their contribution was most apparent when computational methods were embedded within broader surveillance and IPC workflows rather than used as standalone tools.

The added value of AI/ML was most evident when models were used as enabling layers within surveillance workflows. EHR-linked ML approaches supported the identification of transmission routes in WGS-detected outbreaks [17,18,19,20], while automated bioinformatics platforms improved the reproducibility and speed of genomic outbreak analysis [21]. In rapid typing studies, ML-based classification of FTIR, MALDI-TOF MS, SERS, or related spectral data supported preliminary clone screening and prioritization for genomic confirmation [25,26,27,28,29,30,31,32,33]. In prediction studies, ML models based on AST phenotypes or WGS-derived features contributed to lineage prediction, AMR phenotype prediction, and risk-factor analysis [24,34,35,36,37,38,39,40]. These applications suggest that the main contribution of AI/ML in AMR outbreak surveillance is operational rather than standalone: helping laboratories and IPC teams prioritize isolates, classify high-risk clones, automate interpretation, and extend surveillance when universal WGS is not feasible. This interpretation is consistent with the broader genomic surveillance literature highlighting the importance of high-risk lineage monitoring for AMR pathogens such as *E. coli*, *P. aeruginosa*, carbapenem-resistant *A. baumannii*, and other clinically relevant clones [55,56,57,58].

From an implementation perspective, the key issue is not only the accuracy of AI-assisted AMR outbreak surveillance, but also how its outputs are incorporated into routine IPC workflows. Real-world deployment requires predefined procedures indicating when alerts are generated, who reviews them, and how they trigger actions such as additional screening, isolation, environmental investigation, device assessment, or sequencing confirmation. These systems should therefore be implemented with multidisciplinary oversight and pilot testing, ensuring that computational outputs are timely, interpretable, and actionable for IPC teams [17,18,19,20,59,60].

Because several AI-assisted surveillance approaches rely on the integration of genomic data with EHR-derived exposures, patient movement, and clinical metadata [17,18,19,20], data governance, privacy, and regulatory requirements should be considered from the earliest implementation stages. In practice, AI-generated alerts should remain interpretable, securely managed, and subject to expert review, particularly when they may influence IPC actions involving patients, wards, devices, or healthcare workers.

In low-resource healthcare systems, implementation may require tiered surveillance models rather than universal WGS, including the use of phenotypic or AST-based approaches when sequencing cannot be applied to all isolates [24]. Rapid typing and targeted sequencing of high-risk isolates may help preserve early-warning capacity while reducing costs and infrastructure requirements [25,26,27,28,29,30,31,32,33]. Low-coverage nanopore-based workflows may also support outbreak reconstruction in settings with limited sequencing infrastructure [44]. In these settings, AI/ML tools should be locally validated, easy to interpret, and integrated into existing laboratory and IPC workflows to avoid increasing operational complexity.

At the same time, WGS-based approaches, including cgMLST and SNP-based analysis when applied, remained the reference framework for high-resolution outbreak confirmation, cluster refutation, and transmission interpretation. Rapid typing and ML-based screening methods were mainly positioned as triage tools, particularly when rapid preliminary assessment was needed or when large numbers of isolates required prioritization [25,26,27,28,29,30,31,32,33]. Their performance was often species-, lineage-, setting-, and database-dependent, and definitive interpretation generally relied on comparison with WGS or cgMLST reference data. This is supported by methodological work showing that rapid spectral typing can be useful for screening but may have limitations in discrimination and transferability across datasets [61]. In practical terms, rapid typing and AI/ML-based classification may accelerate early IPC awareness, but WGS remains central for defining genetic relatedness, distinguishing monoclonal outbreaks from multiple introductions, and supporting high-resolution transmission analysis [62].

A major cross-cutting theme was the transition from isolate-level relatedness toward integrated genomic epidemiology. Several studies combined genomic data with EHR-derived exposures, patient movement, ward-level information, environmental sampling, device-related investigation, plasmid reconstruction, or regional genomic context [17,18,19,20,41,42,43,44,45,46,47,48,49,50,51,52,53,54]. This integration shifts the purpose of genomic surveillance from asking only whether isolates are related to asking which transmission route, reservoir, resistance mechanism, or IPC action is most plausible. In this sense, the most mature applications were those in which genomic data were embedded within epidemiological and IPC workflows, allowing genomic findings to inform targeted screening, environmental investigation, device-focused interventions, or interpretation of regional AMR dissemination. Previous implementation-focused studies similarly emphasize that genomic surveillance is most useful when sequencing outputs are linked to laboratory workflows, clinical metadata, and infection-control decision-making [59,60].

Transmission reconstruction studies further illustrate this shift from genomic description to operational interpretation. Bayesian, stochastic, and phylodynamic approaches were used to infer transmission direction, identify potential spreaders, distinguish single outbreaks from multiple introductions, and estimate outbreak parameters [41,42,43,44]. These methods can add interpretive value when conventional epidemiology is inconclusive, but their outputs depend on sampling completeness, sequencing accuracy, temporal resolution, and the quality of epidemiological metadata. They should therefore be interpreted as decision-support tools within an IPC investigation rather than as standalone evidence of transmission. This distinction is particularly important in healthcare settings, where incomplete sampling, unobserved colonization, environmental reservoirs, and delayed detection may all affect inferred transmission pathways.

The inclusion of plasmid-level and long-read sequencing studies broadens the scope of outbreak surveillance beyond strain relatedness. Several studies showed that carbapenemase dissemination may involve both clonal spread and plasmid-mediated transfer across unrelated lineages or species [51,52]. Long-read sequencing supported more complete characterization of resistance determinants, plasmid replicons, and outbreak-related genomic structures [54]. This is particularly relevant for carbapenemase-producing *Enterobacterales* and other MDRO, where focusing only on core-genome relatedness may underestimate resistance-gene dissemination. This concern is also supported by recent evidence on carbapenemase-producing *E. coli* bloodstream infections, highlighting genomic plasticity, plasmid-mediated dissemination, and high-risk clones such as ST410, ST167, and ST131 as important drivers of international spread and targets for strengthened genomic epidemiology and infection-control surveillance [63]. Plasmid-aware genomic surveillance, therefore, represents an important direction for IPC-oriented AMR monitoring, especially in settings where horizontal gene transfer contributes to the spread of resistance.

From a clinical perspective, the translational value of AI-assisted AMR surveillance ultimately depends on whether earlier detection and interpretation of outbreak signals lead to meaningful patient-centered and IPC outcomes. These may include faster recognition of transmission, earlier implementation of targeted precautions, improved source identification, reduced unnecessary cohorting, and prevention of additional colonization or infection events [17,18,19,20,41,42,43,47,48,49,50,51,52]. This patient-centered perspective provides a practical benchmark for translating surveillance outputs into clinical value.

Despite these advances, several methodological and operational challenges remain. Many AI/ML models were developed in local datasets, specific lineages, or single healthcare networks, limiting their immediate generalizability. Spectral and phenotype-based models may require local calibration and periodic updating as strain populations, resistance mechanisms, and laboratory workflows change. Genomic thresholds for outbreak relatedness also vary across organisms, methods, and settings. Effective implementation, therefore, requires more than analytical accuracy: it depends on timely sequencing, high-quality metadata, interoperable laboratory and clinical information systems, external validation, and clear procedures for translating analytical outputs into IPC decisions. These implementation requirements are consistent with previous genomic-surveillance experiences in healthcare systems, where workflow integration, turnaround time, and diagnostic algorithms strongly influence the practical value of WGS-based surveillance [61,62].

## 5. Limitations and Future Directions

Several limitations should be acknowledged. The search was limited to English-language studies and three bibliographic databases. A formal risk-of-bias assessment was not performed, in keeping with the scoping review design; however, recurrent methodological and implementation-related constraints were summarized at the domain level to support interpretation of evidence maturity. The included studies were heterogeneous in pathogens, healthcare settings, sequencing or typing methods, analytical approaches, and surveillance objectives. Although this heterogeneity is expected in an emerging interdisciplinary field, it limited direct comparison across studies and precluded meaningful quantitative synthesis. In addition, some included studies involved advanced computational genomic epidemiology rather than AI/ML methods in the strict sense.

The evidence base remains uneven. Several studies were retrospective, proof-of-concept, or focused on selected pathogens and outbreak contexts. Prospective real-time evaluations, multicenter external validation, assessment of IPC outcomes, and cost-effectiveness analyses were less frequent. There is also a need for more standardized reporting of genomic-surveillance workflows, metadata integration, model validation, outbreak definitions, and downstream IPC actions. Future research should prioritize studies that evaluate not only whether AI/ML or computational genomic approaches can detect relatedness or predict lineages, but also whether they improve IPC response, reduce transmission, shorten outbreak duration, or support sustainable surveillance at scale. Emerging culture-free approaches, including mNGS- and metatranscriptomics-based machine-learning models, may further expand rapid AMR prediction, but their role in outbreak surveillance still requires validation against WGS-based and epidemiological frameworks [60,64,65].

## 6. Conclusions

AI/ML and advanced computational approaches are increasingly contributing to genomic surveillance of antimicrobial-resistant outbreak clones in healthcare settings. Their main value lies in supporting, scaling, and interpreting WGS-based surveillance rather than replacing it. By enabling automation, prioritization, rapid screening, prediction, transmission modelling, and integration with IPC-relevant metadata, these approaches can help translate genomic surveillance into more actionable infection-control intelligence. Future AMR outbreak surveillance is therefore likely to depend not simply on more sequencing or more AI, but on better integration of genomic data, computational models, epidemiological context, and IPC workflows.

## Figures and Tables

**Table 1:** Population–Concept–Context framework.

PCC Component	Operational Definition
**Population**	Bacterial antimicrobial-resistant pathogens relevant to healthcare-associated transmission, with emphasis on WHO-prioritized bacterial pathogens, multidrug-resistant organisms, high-risk clones, epidemic lineages, and outbreak-associated strains.
**Concept**	AI/ML methods and advanced computational or statistical approaches integrated with WGS/genomic surveillance, rapid typing, or epidemiological and/or clinical data to support early detection, characterization, transmission reconstruction, or investigation of AMR outbreak clones.
**Context**	Healthcare settings and public health genomic surveillance with direct relevance to healthcare-associated transmission, infection prevention and control, outbreak investigation, transmission reconstruction, or early detection of resistant bacterial clones.

**Table 2:** Characteristics of the included studies according to pathogen or resistance profile, setting, study material or sample size, genomic or typing method, AI/ML or computational approach, and relevance to surveillance or infection prevention and control.

Ref.	First Author; Year	Pathogen/ Resistance Profile	Setting	Study Material/ Sample Size	Genomic/ Typing Method	AI/ML or Computational Method	Surveillance/ IPC Relevance
[17]	Sundermann et al., 2021	*P. aeruginosa*	USA; adult tertiary hospital; gastroscope-associated outbreak	882 isolates; 6 outbreak cases; 1 contaminated gastroscope isolate	WGS; SNP-based clustering	EHR-based ML route analysis	Detected hidden outbreak and implicated gastroscope source
[18]	Sundermann et al., 2022	Multiple healthcare-associated pathogens	USA; adult tertiary hospital; hospital-wide surveillance	3165 isolates; 2752 patient isolates; 99 clusters	WGS; cgSNP clustering	EDS-HAT; EHR-linked ML	Detected outbreaks missed by traditional IPC and inferred transmission routes
[19]	Sundermann et al., 2026	Multiple healthcare-associated pathogens	USA; adult tertiary hospital; WGS-detected outbreaks	172 outbreaks; 476 case patients; EHR data from 48,723 patients	WGS; SNP-based outbreak detection	AI algorithm using EHR-derived exposures	Identified transmission routes missed by manual review
[20]	Sundermann et al., 2026	Multiple healthcare-associated pathogens	USA; adult tertiary hospital; real-time genomic surveillance	4723 isolates; 3921 patient isolates; 172 outbreaks	Weekly WGS; SNP-based outbreak detection	Real-time computational surveillance and impact modelling	Supported real-time IPC actions and estimated infections avoided
[21]	Raven et al., 2022	*S. aureus/*MRSA	UK; clinical microbiology/public health laboratory	781 MRSA genomes from 777 patients	WGS; ST, *mec* detection; SNP relatedness	Fully automated bioinformatics platform	Automated confirmation or refutation of MRSA clusters
[22]	Böhne et al., 2026	*K. pneumoniae* complex; mostly wild type; selected ESBL clusters	Germany; tertiary NICU/intermediate care unit	936 patients; 83 isolates; 10 genomic clusters	WGS; MLST; cgMLST; SKA/SNP analysis	Regression; XGBoost; random forest; SHAP	Identified high-risk VLBW infants and supported risk-adapted IPC
[23]	Price et al., 2022	Gram-negative organisms; AMR genes including *bla*_CTX-M_ variants	UK; neonatal unit; routine screening programme	155 isolates from 44 neonates	WGS; MLST; phylogeny; SNP relatedness, AMR genes	Computational genomic epidemiology	Revealed occult transmission and possible AMR gene transfer
[24]	Ross et al., 2026	*E. coli*; ST131 clade C; MDR/ESBL-associated lineage	Norway; national AMR surveillance	2790 genomic BSI isolates; 24,866 BSI and 22,942 UTI AST isolates	WGS-based clade assignment for training/ validation	Random forest and XGBoost using AST phenotypes	Extended surveillance of high-risk lineage where universal WGS was not feasible
[25]	Candela et al., 2025	*K. pneumoniae*; carbapenemase-producing	Spain; multicenter tertiary-hospital clinical collections	365 isolates; 289 included in FTIR-WGS clustering analysis	FTIR spectroscopy; WGS; cgMLST and ST reference typing	PCA; ANN; SVM and random forest spectral classification	First-line typing to support rapid clone screening and prioritize isolates for WGS confirmation
[26]	Vogt et al., 2019	*E. cloacae* complex	Germany; NICU; routine surveillance and suspected outbreak	239 isolates from 24 patients; 53 sequenced isolates; 14 outbreak-period isolates	FTIR spectroscopy; WGS/SNP and MLST reference typing	UPGMA/PCA clustering and ANN-based spectrum classification	Supported rapid typing for NICU surveillance and outbreak triage
[27]	Novais et al., 2024	*K. pneumoniae*; MDR lineages	Portugal and Spain; clinical and surveillance isolate collections	573 isolates; 293 training isolates and 280 validation isolates	ATR FT-IR; KL typing by *wzi* sequencing and WGS reference	Random forest KL-type classifier; PLS-DA submodels for related KL/O types	Same-day automated lineage typing to support outbreak control and public health surveillance
[28]	Cai et al., 2025	*K. pneumoniae*; CRKP; KPC- and NDM-producing	China; multicenter clinical isolate collections	1532 clinical isolates from four institutions	MALDI-TOF MS; qPCR; MLST; KL typing and phylogenetic analysis	Random forest classifiers; SHAP interpretability	Rapid resistance-mechanism screening, with regional model-update requirements
[29]	Lafolie et al., 2015	*E. coli*; ST131; ESBL/MDR-associated high-risk lineage	France; clinical and environmental isolate collection	109 isolates, including 50 ST131 isolates	MALDI-TOF MS spectral typing; MLST reference typing	Quick Classifier and SVM biomarker models	Rapid ST131 screening to support targeted IPC measures
[30]	Zhang et al., 2023	*K. pneumoniae*; different MLST profiles	China; clinical isolate collection	16 strains; 45 SERS spectra per strain	WGS-based MLST and core-genome phylogeny; SERS spectral typing	OPLS-DA and supervised ML classifiers; best performance with SVM	Proof-of-concept for rapid ST screening to support transmission tracing
[31]	Candela et al., 2023	*P. aeruginosa*; MDR ST175 outbreak clone	Spain; tertiary hospital; hematology ward outbreak	67 isolates; 35 WGS-characterized; 32 ASO-PCR validation isolates	PFGE and WGS reference typing; MALDI-TOF MS and FTIR-S spectral typing	ML classifiers applied to MALDI-TOF MS and FTIR-S spectra	Rapid discrimination of outbreak-related isolates and support for real-time clone screening
[32]	Villa et al., 2026	Gram-negative bacteria including *P. aeruginosa*, *K. pneumoniae*, *E. coli*, *E. cloacae* complex and *Stenotrophomonas maltophilia*	France; ICU; prospective transmission surveillance	283 isolates from 135 ICU patients	IR-Biotyper FTIR; cgMLST reference typing	Species-specific spectral clustering thresholds	Rapid exclusion of unrelated isolates and prioritization for genomic confirmation
[33]	Gonçalves et al., 2025	*K. pneumoniae*; carbapenemase-producing	Portugal; district hospital; real-time surveillance	136 CP-*K. pneumoniae* infection isolates	ATR FT-IR; *wzi* sequencing, MLST; PFGE and selected WGS confirmation	Random forest FTIR classification models with model updating	Real-time sublineage typing supporting outbreak detection and IPC actions
[34]	Liu et al., 2025	CRKP; mainly ST14 and ST433 carrying *bla*_NDM-1_	China; pediatric hospital; neonatal units and NICU	64 CRKP isolates from 58 neonates over 8 years	WGS; selected long-read sequencing; cgSNP phylogeny and plasmid analysis	Random forest regression/classification; permutation importance; computational phylogenetic and plasmid analysis	Identified clonal; healthcare-group and plasmid drivers of CRKP persistence and outbreaks
[35]	Sawhney et al., 2022	*S. aureus*/BORSA; MSSA and MRSA	USA; NICU MRSA pseudo-outbreak and comparator isolates	102 *S. aureus* isolates; 101 high-quality WGS assemblies	WGS; core-genome SNP; MLST, *spa*, accessory genome and AMR gene analysis	Random forest classifier using PBP/GdpP features and beta-lactamase phenotype	Refuted clonal MRSA outbreak and improved interpretation of MRSA surveillance results
[36]	Wang et al., 2021	*S. aureus*/MRSA; MDR lineages	China; multisource surveillance	673 isolates from food and human sources	WGS; MLST; *spa*; SCCmec; phylogeny and Bayesian divergence analysis	Supervised ML using genome-derived k-mers; best performance with RBF-SVM	Identified AMR-associated genomic traits and high-risk MRSA lineages for WGS-based surveillance
[37]	Wang et al., 2022	*S. aureus*/MRSA and MSSA with resistance to multiple antimicrobials	China; multicenter clinical isolate collection	466 isolates from bloodstream infection; hospital-acquired pneumonia and intra-abdominal infection studies	WGS; MLST and SCCmec typing; genome-derived k-mers	Random forest; SVM and XGBoost for MIC and resistance-category prediction	Supported WGS-based AMR phenotype prediction and rapid MRSA/resistance surveillance
[38]	Zhuang et al., 2021	*S. aureus*/MRSA with penicillin plus potassium clavulanate susceptibility	China and UK; teaching hospital and external MRSA validation datasets	284 hospital MRSA isolates; 471 Chinese national MRSA isolates; 287 UK MRSA isolates	WGS; *mecA* genotyping; cgMLST allelic profiles	Random forest susceptibility prediction model	Supported cgMLST-based prediction of MRSA susceptibility and lineage-aware AMR surveillance
[39]	Chaki et al., 2026	*S. aureus*/MRSA with β-lactam and methicillin resistance	Global; public genomic surveillance dataset	111,350 public *S. aureus* genomes; linked MIC data where available	WGS assemblies; AMRFinderPlus; MLST; resistance-gene and gene-pattern analysis	Random forest; XGBoost; Elastic Net and PLS for MIC prediction; temporal and lineage analysis	Mapped global β-lactam and *mecA* -mediated resistance dynamics and supported large-scale WGS-based AMR surveillance
[40]	Mauffrey et al., 2024	*S. aureus/*MRSA; ST228 SCCmec-I	Switzerland; tertiary hospital; post-outbreak MRSA surveillance	421 ST228 MRSA isolates; 218 Lausanne outbreak/post-outbreak isolates analyzed for epidemicity	WGS; SNP phylogeny; pan-genome and accessory-genome analysis	Time-scaled haplotypic density, GWAS and random forest models	Identified genomic markers associated with declining epidemicity after a major nosocomial outbreak
[41]	Fujikura et al., 2019	*E. faecium*/VRE; ST17 with *vanA* and *vanB* genotypes	Japan; tertiary hospital; VRE outbreak	23 VRE isolates from patients and environmental surfaces	WGS; MLST; SNP/SNV and INDEL variant analysis	Bayesian transmission reconstruction integrating genomic variants and epidemiological data	Reconstructed transmission routes; identified spreaders and environmental transmission, and supported IPC measures
[42]	Shimizu et al., 2025	*P. aeruginosa*/CRPA; multiple sequence types	Japan; tertiary children’s hospital; suspected CRPA outbreak	25 CRPA isolates from 10 patients and two environmental sites	Hybrid WGS; MLST; ANI/PFGE comparison; AMR gene and variant analysis	Bayesian transmission inference using JUNIPER; comparative lineage and resistance-mechanism analysis	Ruled out monoclonal outbreak; identified multiclonal CRPA emergence and assessed possible local environmental transmission
[43]	Silvotti et al., 2025	*K. pneumoniae*; KPC-producing; ST307 and ST716	Italy; tertiary hospital; neurorehabilitation unit outbreak	19 KPC-*K. pneumoniae* isolates from colonized or infected patients	WGS; ST assignment; SNP phylogeny; AMR and virulence gene profiling	Bayesian discrete-time stochastic transmission models using Outbreaker2	Revealed seven independent introductions, secondary transmission chains and IPC gaps in admission screening and staff training
[44]	Steinig et al., 2022	*S. aureus*; community-associated MRSA ST93-MRSA-IV	Australia and Papua New Guinea; remote community and hospital-linked surveillance	181 *S. aureus* isolates; 159 with matched Illumina reference data	Low-coverage nanopore sequencing; Illumina reference WGS; SNP-based phylogeny	Random forest SNP polishing; Bayesian phylodynamic birth–death skyline models	Enabled low-cost bacterial outbreak reconstruction and estimation of transmission dynamics in settings with limited sequencing infrastructure
[45]	The et al., 2015	*K. pneumoniae*; MDR; ST15; NDM-1 lineage and ST1559 lineage	Nepal; tertiary hospital; high-dependency pediatric wards	90 selected *K. pneumoniae* isolates; 89 sequenced; 412 additional isolates screened by lineage-specific PCR	WGS; MLST, core-genome SNP phylogeny; resistome and comparative genomics	Bayesian phylogenetic analysis; comparative genomic outbreak reconstruction; lineage-specific PCR screening	Resolved two distinct outbreak lineages; revealed pre-outbreak circulation and supported real-time genetic characterization for IPC
[46]	Morgado et al., 2023	*A. baumannii*/CRAB; XDR IC2/ST2	Brazil; tertiary hospital; nosocomial outbreak	16 clinical *A. baumannii* isolates	WGS; ST assignment; core-genome phylogeny; resistome; virulome and capsule typing	Comparative genomic epidemiology using local and public genomes	Identified high-risk IC2/ST2 outbreak sub-lineages and supported regional genomic surveillance of XDR CRAB
[47]	Ding et al., 2026	*A. baumannii*/CRAB; predominantly ST2	China; tertiary hospital; ICU environmental surveillance	43 CRAB-positive patients; 3390 environmental samples; 178 isolates sequenced	WGS; MLST; cgMLST; core-SNP phylogeny and resistance-gene analysis	Prospective genomic epidemiology with spatiotemporal transmission reconstruction	Identified near-patient environmental contamination; airborne isolate diversity and possible inter-ICU transmission events
[48]	Guo et al., 2026	*E. faecium*/VRE; predominantly ST80	China; two hospitals in Guangdong; hospital-acquired outbreak	101 VR-*E. faecium* isolates from 101 patients	WGS; MLST; cgMLST; SNP analysis; resistome and virulence analysis	Comparative genomic epidemiology; PCA/UMAP; MST; time-dated phylogeny; transmission-cluster analysis; PCR marker development	Characterized a high-risk ST80 VR-*E. faecium* outbreak and supported molecular screening for early detection and surveillance
[49]	Song et al., 2026	*A. baumannii*/CRAB; ST2 carrying *bla*_OXA-23_ and *bla*_OXA-66_	China; tertiary hospital; cardiovascular surgical ICU suspected outbreak	6 infected patients; 146 environmental and hand samples; 27 CRAB isolates analyzed	WGS; MLST; core-genome SNP phylogeny; resistance-gene and mobile-element analysis	Genomic epidemiology with SNP-based relatedness and environmental-source assessment	Refuted a single nosocomial outbreak; identified environmental transmission risk points and guided IPC interventions
[50]	Cissé et al., 2026	*P. aeruginosa*; Ceftazidime and meropenem-resistant; ST1320	France; tertiary hospital; duodenoscope-associated transmission	8 patient cases; 23 *P. aeruginosa* isolates from patients and duodenoscopes	Antibiogram, FTIR, WGS; SNP-based relatedness and ST assignment	Integrated microbiological and genomic investigation of device-associated transmission	Confirmed prolonged silent clonal transmission via duodenoscopes and supported enhanced endoscope surveillance and device-focused IPC
[51]	Jalal et al., 2026	*E. coli* and *K. pneumoniae*/CRE BSI isolates; mainly carrying *bla*_NDM-5_	Egypt; pediatric oncology hospital	189 CRE bloodstream isolates from pediatric oncology patients	WGS; MLST; cgMLST; plasmid reconstruction; resistome; virulome and plasmid phylogeny	Integrated genomic and plasmid-level epidemiology with patient-movement analysis	Distinguished clonal spread from plasmid-mediated dissemination and supported plasmid-level surveillance in high-risk oncology settings
[52]	Leder et al., 2025	*Enterobacterales* species/VIM-producing CPE	Germany; tertiary hospital; longitudinal hospital surveillance	43 inpatient episodes with VIM-CPE; 27 hospital-acquired isolates	Short- and long-read WGS; cgMLST; cgSNP and plasmid analysis	Integrated core-genome and plasmid-level genomic epidemiology with patient-movement analysis	Revealed temporally separated transmission events and plasmid-mediated carbapenemase spread, supporting broader IPC investigations beyond direct patient contact
[53]	García-González et al., 2025	*K. pneumoniae*; 3GC- and carbapenem-resistant	Spain; regional genomic surveillance across eight hospitals	1768 local *K. pneumoniae* isolates; 11,967 contextual genomes	WGS; cgMLST; high-resolution SNP analysis; AMR gene and plasmid-context analysis	Regional genomic surveillance with transmission-group analysis and global phylogenetic contextualization	Mapped intra- and inter-hospital transmission patterns and identified ST307 carrying *bla*_CTX-M-15_ as a major regional transmission driver
[54]	Landman et al., 2024	MDRO including CPE; CPPA; CRAB and MRSA	Netherlands; national MDRO surveillance/reference-laboratory setting	356 MDRO isolates, including 69 MRSA isolates; 24 from an outbreak	Nanopore long-read WGS compared with Illumina short-read WGS; MLST; wgMLST; wgSNP; iMLVA; AMR gene and plasmid-replicon detection	Long-read sequencing workflow evaluation with assembler/ basecaller comparison and outbreak-analysis validation	Supported long-read WGS as a feasible approach for MDRO genomic surveillance; resistance-gene detection and outbreak analysis

**Legend: ANI,** average nucleotide identity; **ANN**, artificial neural network; **ASO-PCR**, allele-specific oligonucleotide polymerase chain reaction; **AST**, antimicrobial susceptibility testing; **BORSA**, borderline oxacillin-resistant *S. aureus*; **BSI**, bloodstream infection; **cgMLST**, core-genome multilocus sequence typing; **cgSNP**, core-genome single-nucleotide polymorphism; **CP**, carbapenemase-producing; **CPE**, carbapenemase-producing *Enterobacterales*; **CPPA**, carbapenemase-producing *P. aeruginosa*; **CRAB**, carbapenem-resistant *A. baumannii*; **CRE**, carbapenem-resistant *Enterobacterales*; **CRKP**, carbapenem-resistant *K. pneumoniae*; **CRPA**, carbapenem-resistant *P. aeruginosa*; **ESBL**, extended-spectrum β-lactamase; **FTIR**, Fourier-transform infrared spectroscopy; **GWAS**, genome-wide association study; **IC**, international clone; **iMLVA**, in silico multiple-locus variable-number tandem-repeat analysis; **INDEL**, insertion/deletion; **IR**, infrared; **MALDI-TOF MS**, matrix-assisted laser desorption/ionization time-of-flight mass spectrometry; **MDRO**, multidrug-resistant organism; **MDR**, multidrug-resistant; **MIC**, minimum inhibitory concentration; **MLST**, multilocus sequence typing; **MRSA**, methicillin-resistant *S. aureus*; **MSSA**, methicillin-susceptible *S. aureus*; **MST**, minimum spanning tree; **NDM**, New Delhi metallo-β-lactamase; **NICU**, neonatal intensive care unit; **OPLS-DA**, orthogonal partial least squares discriminant analysis; **PBP/GdpP**, penicillin-binding protein/guanosine diphosphatase phosphodiesterase; **PCA**, principal component analysis; **PCR**, polymerase chain reaction; **PFGE**, pulsed-field gel electrophoresis; **PLS**, partial least squares; **PLS-DA**, partial least squares discriminant analysis; **qPCR**, quantitative polymerase chain reaction; **RBF**, radial basis function; **SCCmec**, staphylococcal cassette chromosome *mec*; **SERS**, surface-enhanced Raman spectroscopy; **SHAP**, SHapley Additive exPlanations; **SKA**, split k-mer analysis; **SNP**, single-nucleotide polymorphism; **ST**, sequence type; **SVM**, support vector machine; **UMAP**, uniform manifold approximation and projection; **UPGMA**, unweighted pair group method with arithmetic mean; **UTI**, urinary tract infection; **VIM**, Verona integron-encoded metallo-β-lactamase; **VLBW**, very low birth weight; **VRE**, vancomycin-resistant enterococci; **wgMLST**, whole-genome multilocus sequence typing; **wgSNP**, whole-genome single-nucleotide polymorphism; **XDR**, extensively drug-resistant.

## Data Availability

No new data were created or analyzed in this study. Data sharing is not applicable to this article.

## Supplementary Materials

The following supporting information can be downloaded at: https://www.mdpi.com/article/10.3390/antibiotics15060599/s1, Supplementary File S1: PRISMA-ScR checklist; Supplementary Table S1: Search strategy.
