# Supplementary material for: Can Artificial Intelligence Transform Early Warning for Antimicrobial-Resistant Outbreak Clones? Approaches, Gaps, and Opportunities: A Scoping Review
Source: Antibiotics (Basel). 2026 Jun 12;15(6):599. doi: 10.3390/antibiotics15060599 (PMC13295586; doi:10.3390/antibiotics15060599)
Supplement: Supplementary file 1 [file antibiotics-15-00599-s001.zip › antibiotics-4371001-supplementary-Table S1.pdf]

Supplementary Table S1. Database-specific search strategies, record counts, and deduplication details. Search date: the final database search was performed on 6 May 2026.

Scientific databases	Search strategy	Hits
**PubMed/MEDLINE**<br>Search n.1<br>Limits: English.<br>2010-2026	("Artificial Intelligence"[Mesh] OR "Machine Learning"[Mesh] OR "Deep Learning"[Mesh] OR "artificial intelligence"[tiab] OR AI[tiab] OR "machine learning"[tiab] OR ML[tiab] OR "deep learning"[tiab] OR "neural network*"[tiab] OR "random forest*"[tiab] OR "support vector machine*"[tiab] OR "gradient boosting"[tiab] OR "anomaly detection"[tiab] OR "predictive model*"[tiab] OR "clustering algorithm*"[tiab] OR "graph neural network*"[tiab]) AND ("Whole Genome Sequencing"[Mesh] OR "whole genome sequencing"[tiab] OR WGS[tiab] OR "genomic surveillance"[tiab] OR "genomic epidemiology"[tiab] OR "genome sequencing"[tiab] OR cgMLST[tiab] OR "core genome MLST"[tiab] OR SNP[tiab] OR "single nucleotide polymorphism*"[tiab] OR phylogen*[tiab] OR pangenom*[tiab] OR resistome[tiab]) AND ("Drug Resistance, Bacterial"[Mesh] OR "antimicrobial resistance"[tiab] OR "antibiotic resistance"[tiab] OR "drug resistant"[tiab] OR "multidrug resistant"[tiab] OR MDR[tiab] OR MDRO[tiab] OR MRSA[tiab] OR "carbapenem-resistant"[tiab] OR "carbapenem resistant"[tiab] OR ESBL[tiab] OR ESKAPE[tiab] OR "Klebsiella pneumoniae"[tiab] OR "Staphylococcus aureus"[tiab] OR "Acinetobacter baumannii"[tiab] OR "Pseudomonas aeruginosa"[tiab] OR "Enterococcus faecium"[tiab] OR "Enterobacter cloacae"[tiab]) AND ("Disease Outbreaks"[Mesh] OR outbreak*[tiab] OR cluster*[tiab] OR clone*[tiab] OR clonal[tiab] OR lineage*[tiab] OR transmission[tiab] OR "transmission chain*"[tiab] OR "healthcare-associated transmission"[tiab] OR nosocomial[tiab] OR "infection control"[tiab] OR surveillance[tiab])	**205**
**PubMed/MEDLINE**<br>Search n.2<br>Limits: English.<br>2010-2026	("whole genome sequencing"[tiab] OR WGS[tiab] OR "genomic surveillance"[tiab] OR "genomic epidemiology"[tiab]) AND ("machine learning"[tiab] OR "artificial intelligence"[tiab] OR algorithm*[tiab] OR automated[tiab] OR "statistical model*"[tiab] OR "predictive model*"[tiab] OR "electronic health record*"[tiab] OR EHR[tiab] OR "patient movement"[tiab] OR "patient location"[tiab] OR "contact network*"[tiab] OR "transmission route*"[tiab]) AND ("healthcare-associated transmission"[tiab] OR "hospital transmission"[tiab] OR nosocomial[tiab] OR outbreak*[tiab] OR cluster*[tiab] OR transmission[tiab] OR "infection control"[tiab] OR "infection prevention"[tiab]) AND (bacteria*[tiab] OR pathogen*[tiab] OR "Klebsiella pneumoniae"[tiab] OR "Staphylococcus aureus"[tiab] OR MRSA[tiab] OR "Acinetobacter baumannii"[tiab] OR "Pseudomonas aeruginosa"[tiab] OR Enterobacterales[tiab] OR "Enterococcus faecium"[tiab])	**428**
**PubMed/MEDLINE**<br>Search n.3<br>Sensitivity search<br>Limits: English.<br>2010-2026	("Machine Learning"[Mesh] OR "Artificial Intelligence"[Mesh] OR "Deep Learning"[Mesh] OR "machine learning"[tiab] OR "artificial intelligence"[tiab] OR "deep learning"[tiab] OR "random forest*"[tiab] OR "support vector machine*"[tiab] OR "neural network*"[tiab] OR "gradient boosting"[tiab] OR "graph neural network*"[tiab]) AND ("Whole Genome Sequencing"[Mesh] OR "whole genome sequencing"[tiab] OR WGS[tiab] OR genome*[tiab] OR genomic[tiab] OR pangenom*[tiab] OR pan-genom*[tiab] OR resistome[tiab] OR k-mer*[tiab] OR SNP[tiab] OR "single nucleotide polymorphism*"[tiab]) AND ("Drug Resistance, Bacterial"[Mesh] OR "antimicrobial resistance"[tiab] OR "antibiotic resistance"[tiab] OR "drug resistance"[tiab] OR "resistance phenotype*"[tiab] OR "minimum inhibitory concentration"[tiab] OR MIC[tiab]) AND (predict*[tiab] OR classification[tiab] OR model*[tiab] OR "feature selection"[tiab] OR "genomic feature*"[tiab])	**652**

**Scopus**<br>Limits: English.<br>2010-2026	TITLE-ABS-KEY (("artificial intelligence" OR "machine learning" OR "deep learning" OR "neural network*" OR "random forest*" OR "support vector machine*" OR "gradient boosting" OR "anomaly detection" OR "predictive model*" OR "clustering algorithm*" OR "graph neural network*") AND ("whole genome sequencing" OR wgs OR "genomic surveillance" OR "genomic epidemiology" OR"genome sequencing" OR cgmlst OR "core genome mlst" OR snp OR "single nucleotide polymorphism*"OR phylogen* OR pangenom* OR resistome) AND ("antimicrobial resistance" OR "antibiotic resistance"OR "drug resistant" OR "multidrug resistant" OR mdr OR mdro OR mrsa OR "carbapenem-resistant" OR"carbapenem<br>resistant" OR esbl OR eskape) AND (outbreak* OR cluster* OR clone* OR clonal ORlineage* OR transmission O R "transmission chain*" OR "healthcare-associated transmission" ORnosocomial OR "infection control" OR surveillance))	**364**
**Web of Science**<br>Limits: English.<br>2010-2026	TS=(<br>(<br>"whole genome sequencing" OR "whole-genome sequencing" OR WGS OR<br>"genomic surveillance" OR "genomic epidemiology" OR<br>cgMLST OR "core genome multilocus sequence typing" OR<br>"core genome MLST" OR "SNP phylogen*" OR phylogenomic*<br>)<br>AND<br>(<br>"artificial intelligence" OR "machine learning" OR "deep learning" OR<br>"neural network*" OR "random forest" OR "support vector machine" OR<br>SVM OR XGBoost OR "gradient boosting" OR<br>"predictive model*" OR "anomaly detection" OR algorithm*<br>)<br>AND<br>(<br>"antimicrobial resistance" OR "antibiotic resistance" OR<br>"drug resistance" OR AMR OR<br>"multidrug resist*" OR MDR OR<br>"carbapenem resist*" OR "methicillin resist*" OR<br>"vancomycin resist*" OR MRSA OR VRE OR ESBL OR CRE OR CRKP OR CRAB<br>)<br>AND<br>(<br>outbreak* OR cluster* OR clone* OR lineage* OR<br>"high-risk clone*" OR "epidemic clone*" OR<br>"transmission" OR "transmission chain*" OR<br>nosocomial OR hospital* OR healthcare OR<br>"healthcare-associated" OR "infection control"<br>)	**193**

	AND<br>(<br>bacteria* OR bacterial OR pathogen* OR<br>"Klebsiella pneumoniae" OR "Pseudomonas aeruginosa" OR<br>"Acinetobacter baumannii" OR "Staphylococcus aureus" OR<br>"Enterococcus faecium" OR "Escherichia coli" OR<br>Enterobacterales OR Enterobacteriaceae<br>)<br>)<br>NOT TS=(<br>tuberculosis OR "Mycobacterium tuberculosis" OR malaria OR<br>virus OR viral OR SARS-CoV-2 OR COVID-19 OR<br>wastewater OR soil OR aquaculture OR poultry OR cattle OR swine OR pig<br>)	
**Web of Science**<br>Sensitivity search<br>Limits: English;<br>2010-2026	TS=(<br>(<br>"genomic surveillance" OR "whole genome sequencing" OR WGS OR "healthcare-associated transmission" OR "genomic epidemiology"<br>)<br>AND<br>(<br>"artificial intelligence" OR "machine learning" OR "automated" OR algorithm* OR "electronic health record*" OR EHR OR "transmission route*" OR "chart review"<br>)<br>AND<br>(<br>outbreak* OR transmission OR "infection control" OR hospital* OR healthcare<br>)<br>AND<br>(<br>"antimicrobial resistance" OR AMR OR "multidrug resistant" OR MDR OR MRSA OR VRE OR CRE OR CPE OR CRKP OR CRAB OR "carbapenemase-producing"<br>)<br>)	398 screened.<br>**1** additional eligible record

Deduplication step	Records
PubMed/MEDLINE Search n.1	205
PubMed/MEDLINE Search n.2	428
PubMed/MEDLINE Search n.3	652
**Total raw PubMed/MEDLINE records**	**1,285**
Duplicate PubMed/MEDLINE records removed	173
**PubMed/MEDLINE records after within-database deduplication**	**1,112**
Scopus records	364
Web of Science records included in final count, 193 main searches + 1 additional eligible sensitivity-search record.	194
**Total main database records before final deduplication**	**1,670**
Duplicate records removed after merging databases	268
**Records considered after deduplication and additional identification**	**1,402**

Deduplication method. PubMed/MEDLINE records were first deduplicated within PubMed using PMID. After merging PubMed/MEDLINE, Scopus, and Web of Science records, final duplicate removal was performed using DOI, PMID when available, and normalized article title.

PRISMA reporting note. The Web of Science count included 193 records from the main search and one additional eligible record identified through the Web of Science sensitivity search. Overall, 1,843 records were identified from PubMed/MEDLINE, Scopus, and Web of Science. After removing 441 duplicate records, 1,402 records remained for title and abstract screening.
